# Acute Traumatic Injury of the Larynx

**DOI:** 10.1155/2015/393978

**Published:** 2015-03-03

**Authors:** K. O. Kragha

**Affiliations:** Department of Radiology, University of Louisville, 530 S. Jackson Street, CCB C07, Louisville, KY 40202, USA

## Abstract

Laryngeal trauma is rare but serious and potentially deadly injury. The prompt diagnosis and management of acute laryngeal trauma is necessary because the clinical presentation is variable depending on the location, severity, and mechanism of injury. Two case histories are presented: (1) case history A: a 53-year-old male, after motor vehicle accident, fractured the mid anterior thyroid cartilage and both aspects of the cricoid cartilage; however, this patient was asymptomatic from the above fractures; and (2) case history B: a 41-year-old male who sustained trauma to the chest, neck, and left arm after being struck by a large lead pipe which fractured the left aspect of the cricoid cartilage was symptomatic. The type rather than the severity of acute laryngeal injury and the mechanism of injury may be related to symptomatology. Acute laryngeal trauma should be recognized by trauma radiologists and emergency room physicians. Early diagnosis and management of acute laryngeal trauma may prevent unnecessary specialty consults and long-term complications.

## 1. Introduction

Laryngeal trauma is rare but potentially deadly injury. Laryngeal trauma is classified as either penetrating or blunt and supraglottic, glottic, or infraglottic. Laryngeal trauma may heal with fibrous union, deformity, and altered laryngeal function even after minor trauma [1–9]. Features of laryngeal trauma include loss of normal anatomic landmarks, tenderness, crepitus, soft tissue emphysema, dysphonia, aphonia, laryngeal obstruction, dyspnea, stridor, hoarseness, neck pain, hemoptysis, dysphagia, and odynophagia [5].

The purpose of this report is to promote prompt diagnosis and management of acute laryngeal trauma by trauma radiologists and emergency room physicians because it prevents long-term complications. Delayed treatment of acute laryngeal trauma results in poorer outcome than early intervention in acute laryngeal injury. Butler et al. [7] reported that a delayed treatment group after acute laryngeal trauma had about 28% good voice outcome and about 73% had good airway function whereas early treatment group after acute laryngeal injury had about 78% good voice outcome and about 93% good airway function. Ninety-nine percent of all their patients had normal deglutition.

## 2. Report of Cases

**Figure 1:**
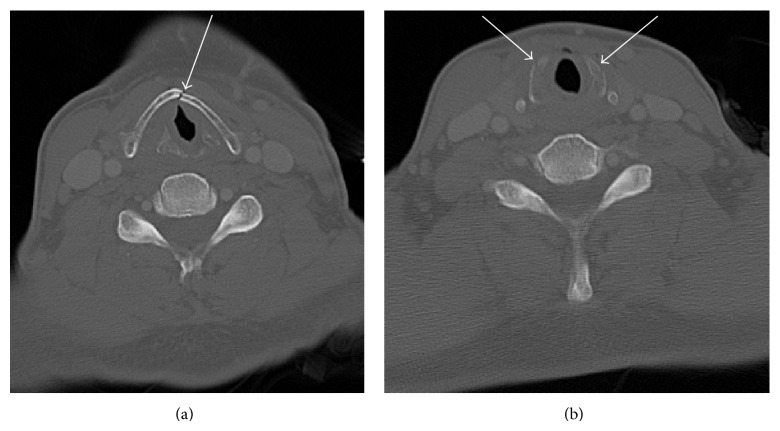
(a) Case A: axial contrast CT of the neck shows acute fracture of the mid anterior thyroid cartilage. (b) Case A: axial contrast CT of the neck shows acute comminuted fracture of both aspects of the cricoid cartilage.

*Case History A*. A 53-year-old male presented to the emergency room after motor vehicle accident. He was a restrained driver going approximately 40 mph. The patient was hemodynamically stable when he presented to the emergency, complaining of chest pain. The patient had CT of the neck that showed acute fracture of the mid anterior thyroid cartilage and acute comminuted fracture of both aspects of the cricoid cartilage (Figures 1(a) and 1(b)). The patient's comorbidities related to the acute laryngeal injury include the following: pneumomediastinum, air in the neck, retropharyngeal air, bilateral rib fractures, left femoral head dislocation, and left acetabular fracture. The patient's acute laryngeal injury was managed conservatively. He was initially nothing by mouth but was cleared for regular food and started on a diet after a normal esophagram. However, the patient was asymptomatic from the above fractures.

**Figure 2:**
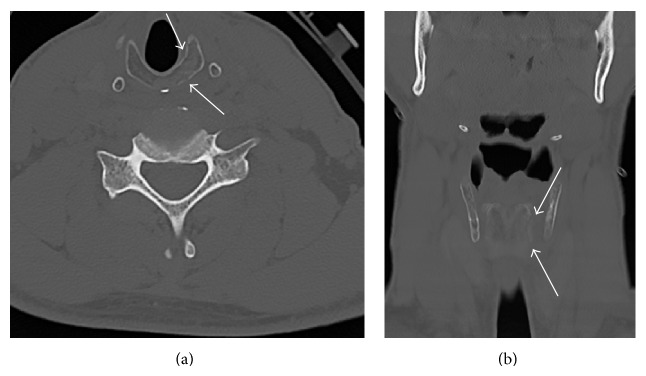
(a) Case B: axial noncontrast CT of the cervical spine shows acute comminuted fracture of the left aspect of the cricoid cartilage. (b) Case B: coronal noncontrast CT of the cervical spine shows acute fracture of the left aspect of the cricoid cartilage.

*Case History B*. A 41-year-old male presented to the emergency room after being struck on the chest, neck, and left arm by a large lead pipe. The patient had CT of the cervical spine that showed acute comminuted fracture of the left aspect of the cricoid cartilage (Figures 2(a) and 2(b)). The patient had difficulty breathing initially, voice changes including cracking and increased raspiness of his throat, dysphonia, and tenderness over palpation of the thyroid cartilage. He had no stridor or crepitus. The patient had left arytenoid hematoma with left cord paresis or paralysis. The patient's comorbidity related to the acute laryngeal injury is right radial fracture. The patient's acute laryngeal injury was managed conservatively. He was kept overnight for monitoring, placed on Decadron, and kept on nothing by mouth overnight. He did well and was started on a diet.

## 3. Discussion

Acute laryngeal trauma may present with dyspnea, paralysis of the vocal cord, airway obstruction due to vocal cord paralysis, loss of normal anatomic landmarks, tenderness in the neck, crepitus, and soft tissue emphysema, dysphonia, aphonia, stridor, hoarseness, very rough voice, neck pain, hemoptysis, dysphagia, and odynophagia [1, 4, 5, 8]. Case A was involved in low speed motor vehicle accident whereas case B was assaulted with a large lead pipe. Case A who had obvious injury by CT was asymptomatic whereas case B who had more subtle injury by CT was symptomatic. Case B had dyspnea, voice changes including cracking and increased raspiness of his throat, dysphonia, tenderness with palpation of the thyroid cartilage, and left cord paresis or paralysis; he had no stridor or crepitus. This study shows that the type rather than the severity of acute laryngeal injury and the mechanism of injury may be related to symptomatology. However, Butler et al. [7] noted that the severity and type of laryngeal injury are of prognostic value.

Case A had comorbidities of pneumomediastinum, air in the neck, retropharyngeal air, bilateral rib fractures, left femoral head dislocation, and left acetabular fracture related to the acute laryngeal trauma, whilst case B had comorbidity related to the acute laryngeal trauma of right radial fracture. This is in agreement with the findings of Fuhrman et al. [2] who reported that 4 patients out of 18 patients (22.2%) had chest trauma, 3 patients out of 18 (16.7%) each had facial fractures, facial lacerations, long bone fractures, and no comorbidity (isolated injury), and 1 patient out of 18 (5.6%) each had closed head injury and esophageal laceration.

Acute laryngeal trauma is classified into five groups: (1) minor endolaryngeal hematoma or laceration without detectable fracture, (2) edema, hematoma, minor mucosal disruption without exposed cartilage, nondisplaced fracture noted on CT, (3) massive edema, mucosal tear, exposed cartilage, cord immobility, displaced fracture, (4) same as group 3 but with more than two fracture lines or massive trauma to laryngeal mucosa, and (5) complete laryngeal separation [2, 7].

The initial management of acute laryngeal trauma is the establishment of a secure airway via oral intubation, local tracheotomy, or cricothyroidotomy. There are a few management options of acute laryngeal trauma as follows: (1) observation, supportive care including observation, humidified air, supplemental oxygen, voice rest, and elevation of the head of the bed for injuries consisting of minimal mucosal trauma and minor hematoma and no visualized or palpable fractures; (2) observation, supportive care, direct laryngoscopy, and esophagoscopy, if the full extent of acute laryngeal trauma could not be determined; (3) direct laryngoscopy and esophagoscopy, open surgical repair, for injuries that consist of vocal cord immobility, significant mucosal lacerations, cartilage exposure, multiple and/or displaced fractures, cricoid fractures, disruption of the cricoarytenoid joint, injury to the anterior commissure, or laceration of the free edge of the vocal cord; and (4) direct laryngoscopy and esophagoscopy, open surgical repair with stent placement for injuries that consist of comminuted cartilaginous fractures, massive mucosal trauma or injuries involving the anterior commissure [2, 3, 7].

The diagnosis of acute laryngeal injury is difficult and may be missed even by experienced radiologists. If acute laryngeal injury is suspected 2D and 3D CT reconstructions may provide additional diagnostic utility to multiaxial CT imaging [9]. In this study there were no 2D and 3D reconstructions.

## 4. Summary

The type rather than the severity of acute laryngeal injury and the mechanism of injury may be related to symptomatology.
